# FGF-23 protects cell function and viability in murine pancreatic islets challenged by glucolipotoxicity

**DOI:** 10.1007/s00424-022-02772-x

**Published:** 2022-11-28

**Authors:** Betina Pajaziti, Kenneth Yosy, Olga V. Steinberg, Martina Düfer

**Affiliations:** Institute of Pharmaceutical and Medicinal Chemistry, Dept. of Pharmacology, University of Münster, Corrensstraße, 48, 48149 Münster, Germany

**Keywords:** Beta-cell, Calcineurin, Glucolipotoxicity, FGF-23, Insulin, JNK

## Abstract

The fibroblast growth factor FGF-23 is a member of the FGF-15/19 subfamily with hormonal functions. Besides its well-known role for bone mineralization, FGF-23 is discussed as a marker for cardiovascular disease. We investigated whether FGF-23 has any effects on the endocrine pancreas of mice by determining insulin secretion, electrical activity, intracellular Ca^2+^, and apoptosis. Acute application of FGF-23 (10 to 500 ng/ml, i.e., 0.4 to 20 nM) does not affect insulin release of murine islets, while prolonged exposure leads to a 21% decrease in glucose-stimulated secretion. The present study shows for the first time that FGF-23 (100 or 500 ng/ml) partially protects against impairment of insulin secretion and apoptotic cell death induced by glucolipotoxicity. The reduction of apoptosis by FGF-23 is approximately twofold higher compared to FGF-21 or FGF-15/19. In contrast to FGF-23 and FGF-21, FGF-15/19 is clearly pro-apoptotic under control conditions. The beneficial effect of FGF-23 against glucolipotoxicity involves interactions with the stimulus-secretion cascade of beta-cells. Electrical activity and the rise in the cytosolic Ca^2+^ concentration of islets in response to acute glucose stimulation increase after glucolipotoxic culture (48 h). Co-culture with FGF-23 further elevates the glucose-mediated effects on both parameters. Protection against apoptosis and glucolipotoxic impairment of insulin release by FGF-23 is prevented, when calcineurin is inhibited by tacrolimus or when c-Jun N-terminal kinase (JNK) is blocked by SP600125. In conclusion, our data suggest that FGF-23 can activate compensatory mechanisms to maintain beta-cell function and integrity of islets of Langerhans during excessive glucose and lipid supply.

**Supplementary information:**

The online version contains supplementary material available at 10.1007/s00424-022-02772-x.

## Introduction

Worldwide, the number of patients diagnosed with type 2 diabetes mellitus (T2DM) is rapidly increasing. Recent estimations suggest that diabetes prevalence will rise to 629 million in 2045 [18]. It is well established that nutrient excess and obesity are crucial risk factors. In search for new therapeutic options, fibroblast growth factors (FGFs) are in the focus of interest. The family of FGFs consists of 22 proteins involved in numerous processes regulating proliferation and development of tissues. Among them, FGF-15/19, FGF-21, and FGF-23 hold an exceptional position, as they are not only growth factors but also act as hormones. While there are several reports about the effects of FGF-15/19 and FGF-21 on glucose homeostasis [19, 39, 52], little is known about the role of FGF-23 in this context. FGF-23 is an important regulator for vitamin D_3_ and phosphate signaling. Its main functions are to decrease 1,25-dihydroxy-vitamin D_3_ plasma concentration and to increase elimination of phosphate [8]. Meanwhile, this spectrum has been extended by several non-renal effects of FGF-23, including alterations of heart morphology, endothelial function, vascular calcification, and inflammatory processes [21]. The action of FGF-23 depends on the target tissue and on the health status of the organism. Elevated FGF-23 plasma concentration was shown to be correlated to left ventricular hypertrophy in patients with chronic kidney disease [16]. By contrast, local secretion of FGF-23 seems to protect vascular smooth muscle cells against phosphate-induced calcification [55]. Besides transcriptional regulation via vitamin D_3_ and parathyroid hormone, expression of FGF-23 can be induced by leptin [50] pointing to a crosstalk between FGF-23, nutrition, and energy balance. Recently, altered plasma concentrations of FGF-23 were linked to insulin resistance and obesity [22, 53]. For obese adolescents, an inverse correlation of FGF-23 and insulin resistance was determined [29, 53]. By contrast, obesity and insulin resistance are associated with elevated FGF-23 plasma concentrations in adults [17, 34]. An increase in circulating FGF-23 in response to augmented fat intake is also reported for mice [20]. FGF-23 interacts with FGF receptor (FGFR) 1c, 3, and 4. Binding affinity to FGFRs can be enhanced by the co-factor klotho [28, 36], but klotho-independent effects are also described [9, 16, 37]. Expression of FGFR1c and alpha-klotho was detected in mouse beta-cells and human pancreatic islets [23, 32] suggesting that, in principle, FGF-23 can activate intracellular signaling pathways in beta-cells.

As the direct effects of FGF-23 on insulin secretion are unknown, the aim of our in vitro study was to elucidate whether this FGF influences stimulus-secretion coupling and cell viability of pancreatic islets and beta-cells under physiological and pathophysiological conditions.

## Research design and methods

### Isolation and culture of pancreatic islets and islet cells

Islets of Langerhans were isolated from adult male or female C57BL/6 N mice (Charles River, and animal facility of the Dept. of Pharmacology, Münster, Germany). The principles of laboratory animal care were followed according to German laws (Az. 39.32.7.1 and 53.5.32.7.1/MS-12668). Mice were euthanized by CO_2_. Islets were isolated by collagenase digestion (Collagenase P, Roche Diagnostics) and cultured at 37 °C in 5% CO_2_ humidified atmosphere. For investigation of apoptosis, the islets were trypsinized to obtain clusters and single cells.

Islets or dispersed islet cells were allowed to recover in RPMI 1640 culture medium (11.1 mM glucose) supplemented with 10% fetal calf serum, 100 U/ml penicillin, and 100 μg/ml streptomycin (Fisher Scientific) overnight. The next day, the standard medium was supplemented with FGFs or other test substances as indicated. For glucolipotoxic conditions, 100 μM palmitate, bound to bovine serum albumin, was added to the culture medium and glucose concentration was elevated to 25 mM in all experimental setups except for determination of apoptosis, where 500 μM albumin-bound palmitate and 33 mM glucose were used.

### Insulin secretion and content

For long-term investigations, islets were cultured with the indicated compounds for 48 h. Culture medium was changed to buffer solution (without any test compounds) containing 5.6 mM glucose (2 h), followed by 3 mM glucose (1 h) to gradually decrease insulin secretion to a basal level. Thereafter, batches of five islets were incubated at 37 °C for 1 h (glucose concentration as indicated in the respective experiment) to determine insulin release. To test for any acute effects of FGF-23, FGF-21, or FGF-15/19, the compounds were added directly before the 1-h steady-state incubation. For determination of insulin content, the supernatant was removed and islets were lysed by acidic ethanol. Insulin was quantified by a radioimmunoassay (Rat insulin RIA, Merck Millipore).

### TUNEL assay

A TUNEL assay (in situ cell death detection kit, fluorescein, Roche Diagnostics) was performed according to the manufacturer’s protocol for determination of apoptotic cell death. Islet cells were cultured under control (48 h or 7 d) or glucolipotoxic (7 d) conditions in the presence or absence of FGFs and other test substances. Islet cells were fixed (3% paraformaldehyde), permeabilized (0.1% Triton-X), and stained according to the manufacturer’s protocol. Apoptosis was monitored by changes in fluorescein fluorescence (480 nm). The total number of cells was determined by bisbenzimide (Hoechst 33258) staining (380 nm). Approximately 200 to 400 cells were counted per preparation for each condition.

### Electrophysiology

Electrical activity of whole islets was determined by extracellular membrane potential recordings with microelectrode arrays (MEA2100-system, 60MEA200/30iR-Ti, MC-Rack software, Multi Channel Systems). Islets were cultured under control or glucolipotoxic conditions in the presence or absence of FGF-15/19, FGF-21, or FGF-23 for 48 h as indicated in the respective experiments. After culture, the islets were transferred to the recording chamber; medium was changed to bath solution containing 3 mM glucose (without FGFs) to terminate electrical activity. Thereafter, islets were stimulated by elevating glucose concentration to 8 or 10 mM.

### Intracellular Ca^2+^ concentration ([Ca^2+^]_c_)

Islets were incubated on poly-l-lysine-coated cover slips with or without FGF-23 in control or glucolipotoxic medium for 48 h. For determination of [Ca^2+^]_c_, islets were loaded with fura-2 acetoxymethylester (fura-2 AM, 5 μM, 37 °C, 30 min). Medium was replaced by bath solution without FGF-23 during loading with fura-2 AM and recording of [Ca^2+^]_c_. Fluorescence was excited at 340 nm and 380 nm and emission was measured by a digital camera (filter 515 nm).

### Solutions and chemicals

For determination of insulin release the following buffer solution was used (mM): 122 NaCl, 4.7 KCl, 1.1 MgCl_2_, 2.5 CaCl_2_, 0.5% BSA, and 10 HEPES (pH 7.4). [Ca^2+^]_c_ experiments were performed at 37 °C in a solution of pH 7.4 containing (mM): 140 NaCl, 5 KCl, 1.2 MgCl_2_, 2.5 CaCl_2_, and 10 HEPES. Glucose was added as indicated. Test compounds were either added directly to bath solution (acute effects of FGFs on insulin secretion) or to the culture medium for a period of 48 h or 7 days (all other experiments). Glucolipotoxic medium contained 25 mM glucose and 100 μM palmitate or 33 mM glucose and 500 μM palmitate. Briefly, palmitate was dissolved in 0.1 N NaOH and further diluted in double-distilled water containing 0.56% or 2.8% fat-free bovine serum albumin. This solution was added to the culture medium in a proportion of 1:10 to obtain the final palmitate concentration of 100 μM or 500 μM. The control medium contained the respective amount of fat-free bovine serum albumin and 0.1 N NaOH. Fura-2 AM, rat insulin and mouse recombinant FGF-15 (referred to as FGF-15/19 in the text), FGF-21, and FGF-23 were ordered from Biotrend. SP600125 and tacrolimus were from Selleckchem, PD-161570 from Sigma-Aldrich and PD-173074 from Biomol. All other chemicals were from Sigma-Aldrich or Diagonal.

### Data evaluation and statistics

Data were collected from islets or islet cells of at least three independent preparations for each series of experiments. Mean [Ca^2+^]_c_ and area under the curve (AUC) of Ca^2+^ were assessed by averaging the last 5 min before changing glucose concentration after subtracting the basal Ca^2+^ concentration. For evaluation of electrical activity, the fraction of plateau phase (FOPP, time with spike activity in relation to the total interval) was calculated for a period of 20 min. Apoptosis was determined by counting the number of TUNEL-positive cells in relation to all cells in 10 randomly selected fields of each sample. All values are given as means ± SD. Statistical significance was assessed by Student’s *t*-test (Fig. 2a and Suppl. Figure 2b) or ANOVA followed by Student–Newman–Keuls post hoc test for multiple comparisons. Values of *p* < 0.05 were considered as significantly different.

## Results

### Glucose-stimulated insulin secretion is negatively influenced by the endocrine FGFs under standard conditions

**Fig. 1:**
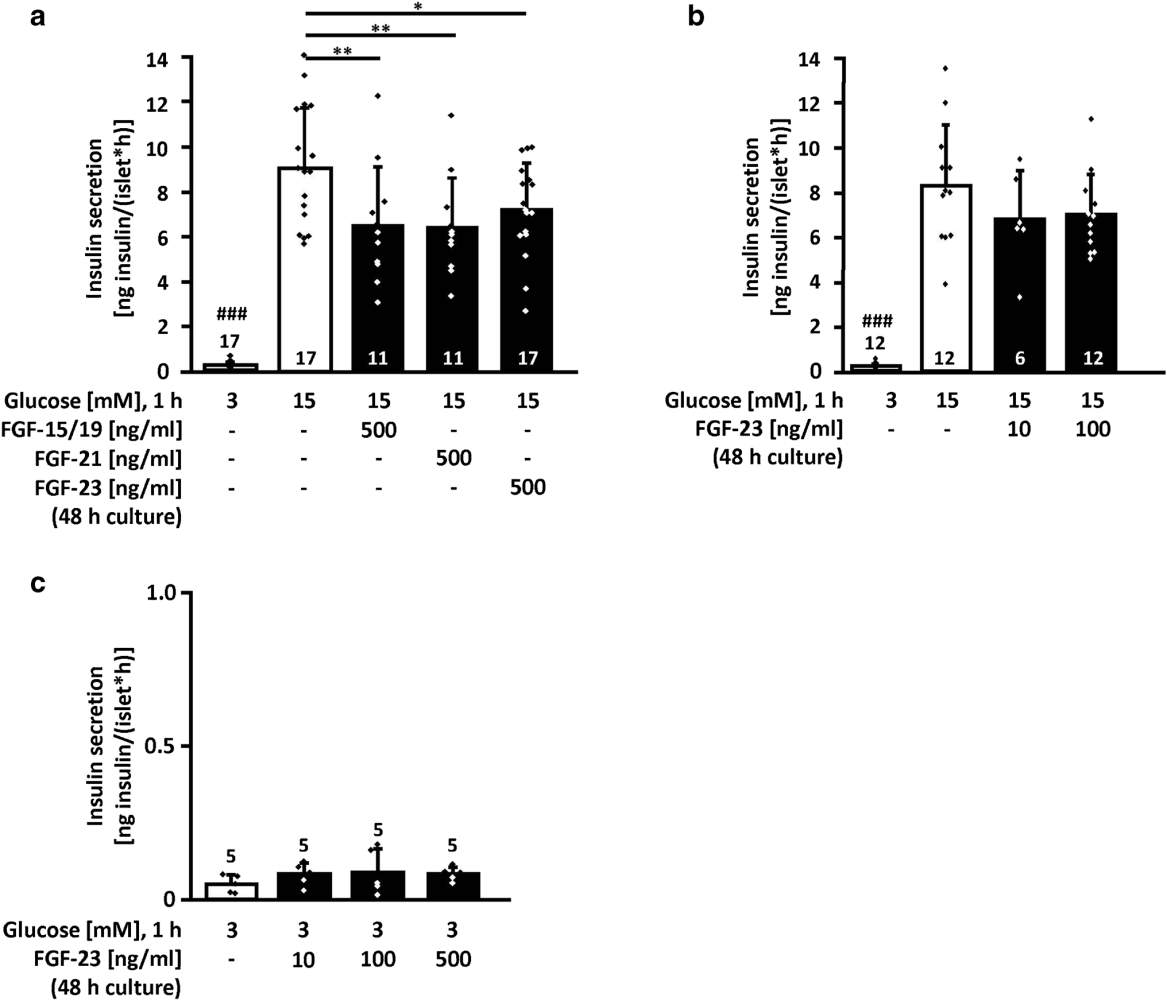
FGF-23 lowers glucose-stimulated insulin secretion under standard conditions. **a** Steady-state incubation (1 h) of murine islets was performed after culturing islets with or without 500 ng/ml FGF-15/19, FGF-21, or FGF-23 under standard conditions for 48 h. All three FGFs lowered glucose-stimulated insulin release. **b** Insulin secretion stimulated by 15 mM glucose also showed a tendency to decline when exposed to 10 or 100 ng/ml FGF-23 for 48 h, whereas **c** basal secretion (3 mM glucose) was unaffected by 10, 100, and 500 ng/ml FGF-23. In these experiments, FGF-23 was not present during the acute determination of glucose-stimulated insulin secretion. Numbers in bars indicate the number of independent islet preparations; *p < 0.05, **p < 0.01, ^###^*p* < 0.001 vs. all other conditions

As it is not known whether FGF-23 directly influences pancreatic islets, glucose-stimulated insulin release (1 h, steady-state incubation) was analyzed after treatment of murine islets with FGF-23 for 48 h. For comparison, we also tested the other two members of the endocrine FGF-family, FGF-15/19, and FGF-21. Surprisingly, insulin secretion in response to acute stimulation with 15 mM glucose was decreased by all three FGFs at a concentration of 500 ng/ml, which is in the low nanomolar range (FGF-15/19 and FGF-23: ~ 20 nM, FGF-21: 23 nM) (Fig. 1a). The inhibitory effect of FGF-23 was slightly less pronounced compared to FGF-15/19 and FGF-21. Glucose-stimulated insulin secretion tended to be decreased after 48-h culture with lower FGF-23 concentrations (10 or 100 ng/ml), but the effect neither reached statistical significance nor showed any dose-dependency (Fig. 1b). In contrast, basal insulin secretion of isolated mouse islets was similar to control after 48-h culture with 10, 100, or 500 ng/ml FGF-23 (Fig. 1c). In addition, we tested whether FGF-23 has any acute effect on insulin secretion. Acute administration of FGF-23 for 1 h did not affect glucose-stimulated insulin release (Suppl. Figure 1).

### Endocrine FGFs have different effects on electrical activity and islet cell viability under standard conditions

**Fig. 2:**
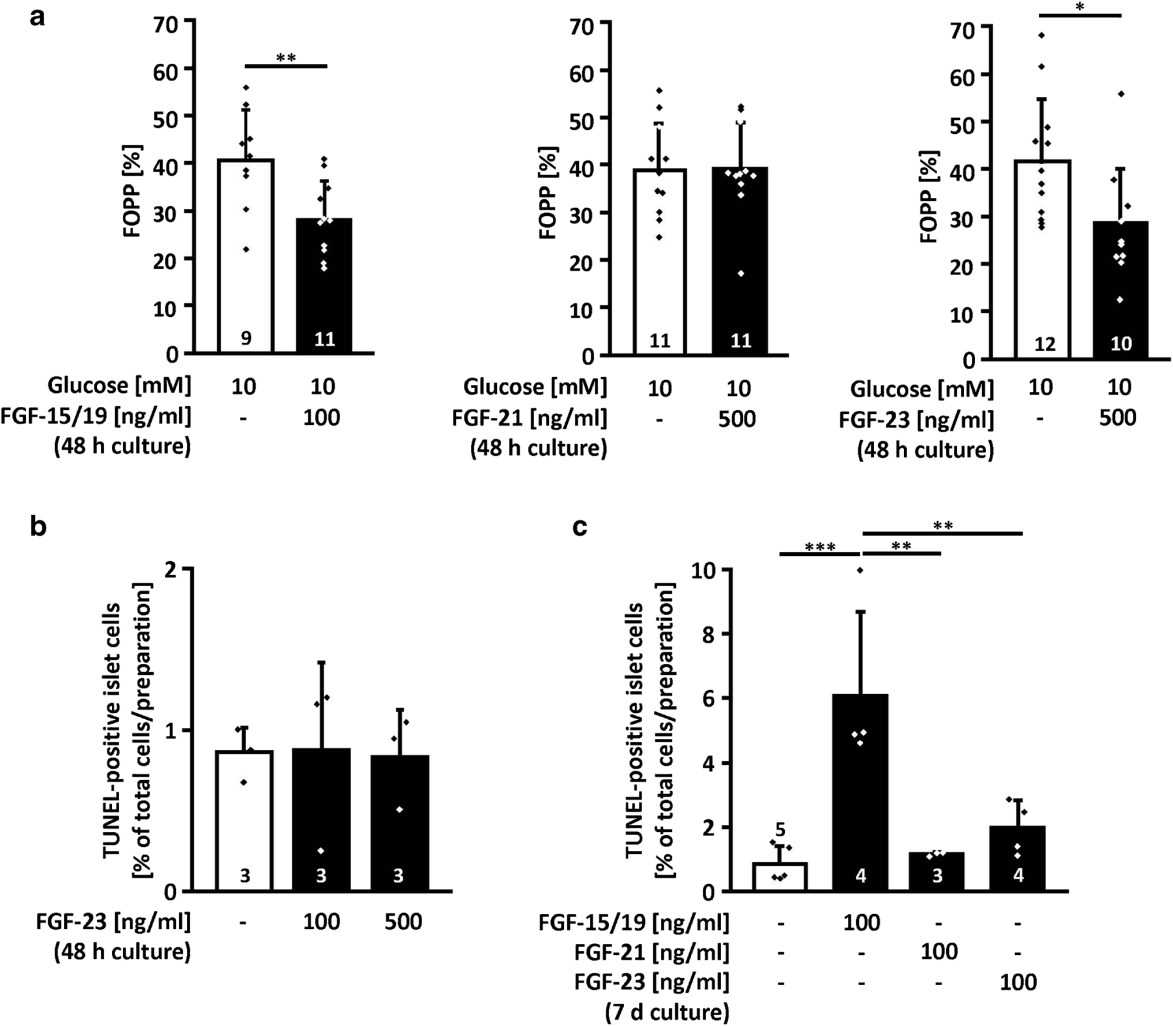
FGF-23, FGF-21, and FGF-15/19 differently affect electrical activity and islet cell mass under standard conditions. **a** The time with electrical activity (fraction of plateau phase, FOPP) was determined by extracellular recordings of islets on MEA chips after culture (48 h) with or without FGF-15/19, FGF-21, or FGF-23. Electrical activity was induced by acute stimulation with 10 mM glucose. The FOPP was decreased to the same level by 100 ng/ml FGF-15/19 or 500 ng/ml FGF-23. Islets treated with 500 ng/ml FGF-21 revealed no changes in the FOPP compared to controls. **b** Apoptotic cell death was low (< 1%) under control conditions (standard medium, 10 mM glucose). Co-culture with 100 or 500 ng/ml FGF-23 (48 h) did not influence cell viability. (c) Islet cells cultured for 7 days in medium supplemented with 100 ng/ml FGF-21 or FGF-23 showed no significant increase in the amount of apoptotic cells, whereas co-culture with 100 ng/ml FGF-15/19 resulted in a significantly elevated fraction of apoptotic cells. Numbers in bars indicate the number of islets (**a**) or independent islet preparations (**b**, **c**); *p < 0.05, **p < 0.01, ***p < 0.001

To elucidate whether the unexpected decrease in insulin release is correlated to a decline in islet cell function or mass, the influence of the endocrine FGFs on the membrane potential and on apoptotic cell death was investigated. The experiments revealed that the 3 members of the FGF-15/19 subfamily influence islet cells differently. For determination of electrical activity, mouse islets were placed on a microelectrode array (MEA) and electrical activity was measured by extracellular recording after 48-h culture with FGF-15/19, FGF-21, or FGF-23. Islets were initially perifused with a bath solution containing 3 mM glucose. Thereafter, glucose was elevated to 10 mM. The fraction of plateau phase (FOPP), i.e., the time where the membrane is depolarized and bursts of Ca^2+^ action potentials occur, was around 40% in glucose-stimulated islets. It was decreased by approximately 13% by 500 ng/ml FGF-23 but not altered by 500 ng/ml FGF-21. FGF-15/19 reduced the FOPP already at a concentration of 100 ng/ml to the level of 500 ng/ml FGF-23 (Fig. 2a).

Culturing islet cells with 100 or 500 ng/ml FGF-23 for 48 h did not elevate the fraction of apoptotic cells (Fig. 2b). After a prolonged culture period of 7 d, 100 ng/ml FGF-15/19 clearly elevated apoptosis (Fig. 2c). FGF-21 and FGF-23 had no significant effect, although it should be noted that there was a tendency towards increased cell death with FGF-23.

### FGF-23 protects against islet cell death and impairment of insulin release under glucolipotoxic conditions

**Fig. 3:**
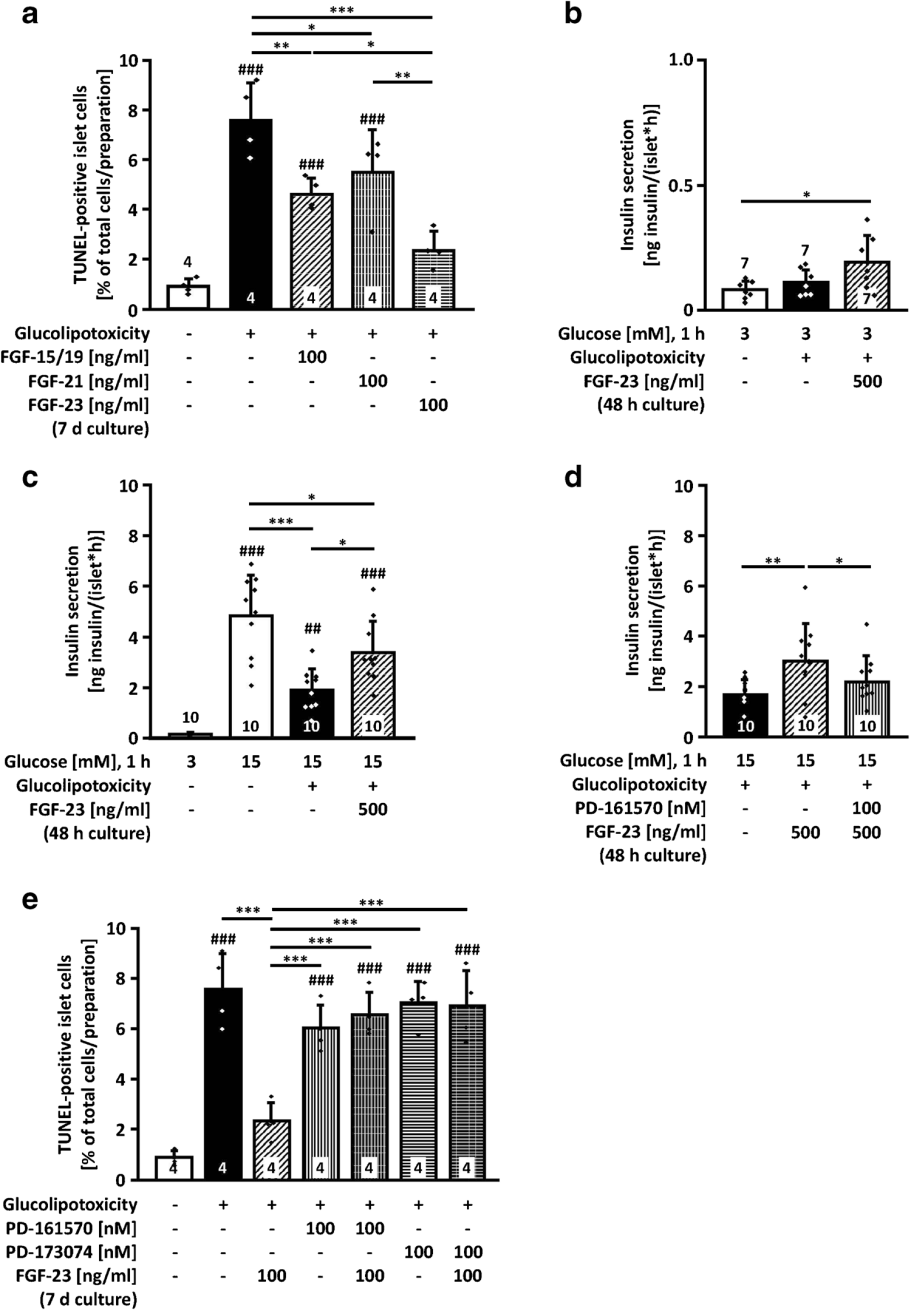
FGF-23 partially protects against impairment of insulin release by glucolipotoxicity and reduces glucolipotoxicity-induced cell death. **a** The number of apoptotic islet cells increased after culture in glucolipotoxic medium for 7 days. FGF-15/19, FGF-21, and FGF-23 (100 ng/ml) prevented the impairment of islet cell viability caused by glucolipotoxicity with FGF-23 leading to the strongest decline. **b** Insulin release in the presence of 3 mM glucose (1 h) was not affected by glucolipotoxicity, but elevated vs. control by the combination of glucolipotoxicity and FGF-23 (500 ng/ml). **c** Glucolipotoxicity reduced glucose-stimulated (15 mM glucose, 1 h) insulin release. FGF-23 partially prevented this effect. **d** Addition of the FGFR inhibitor PD-161570 (100 nM) during glucolipotoxic culture abrogated the protective effect of FGF-23. **e** The protective effect of FGF-23 on glucolipotoxicity-induced apoptotic cell death was prevented by the FGFR inhibitors PD-161570 and PD-173074 (both 100 nM). Glucolipotoxicity: 33 mM glucose, 500 μM palmitate (**a**, **e**); 25 mM glucose, 100 μM palmitate (**b**–**d**). Numbers in bars indicate the number of independent islet preparations; *p < 0.05, **p < 0.01, ***p < 0.001, ##*p* < 0.01, ###*p* < 0.001 vs. control (**a**, **e**) or vs. 3 mM glucose (**c**)

It is well known that progressive impairment of insulin secretion in response to excessive nutrient supply is a key event during the development of T2DM. Therefore, we evaluated the effect of FGF-23 under conditions mimicking the pathophysiologic situation of overnutrition. As an in vitro approach, palmitate in combination with high glucose concentrations was used for induction of cell damage within a relatively short period of time that allowed the monitoring of glucolipotoxic effects in primary mouse islets. Islet cells were exposed to control or glucolipotoxic medium in the presence or absence of the three endocrine FGFs for 7 days. The fraction of apoptotic cells significantly increased after glucolipotoxic culture compared to control conditions (Fig. 3a). The presence of all three FGFs (100 ng/ml) during glucolipotoxic culture significantly reduced apoptosis. The strongest protective effect was observed in glucolipotoxicity-challenged islet cells exposed to FGF-23. To elucidate whether the protective effect of FGF-23 on islet cell mass under glucolipotoxic conditions had an impact on insulin secretion during glucolipotoxicity, glucose-stimulated insulin secretion was measured after culturing islets in glucolipotoxic medium. Figure 3 b demonstrates that glucolipotoxicity per se had no effect on basal insulin release. After co-culture with FGF-23 (500 ng/ml, i.e., ~ 20 nM), the rate of basal secretion increased vs. standard conditions (hatched vs. white bar), whereas the effect was not significant vs. glucolipotoxicity (hatched vs. black bar). Importantly, FGF-23 partially protected against the reduction of glucose-stimulated insulin secretion induced by glucolipotoxicity (Fig. 3c): after 48-h pre-treatment of murine islets in the presence of 25 mM glucose and 100 μM palmitate, the acute secretory response of the islets to stimulation with 15 mM glucose was lowered to approximately 40% compared to controls. Co-culture with FGF-23 significantly improved glucose-induced insulin release (Fig. 3c, hatched vs. black bar), whereby secretion was restored to more than 70% of the control value. To determine whether the partial protective effect of FGF-23 was due to an increase in insulin content, we examined the total amount of insulin in islets. Chronic treatment with glucolipotoxic medium lowered insulin content to 18 ± 12%. Co-culture with 500 ng/ml FGF-23 did not prevent this with regard to absolute values (Suppl. Figure 2a). To consider the variability in insulin content between different mouse preparations, data were also analyzed by normalizing the values to the respective control. This revealed a small protective effect by FGF-23 (Suppl. Figure 2b).

The FGFR inhibitor PD-161570 [46] was added during the culture period of 48 h in another series of experiments. PD-161570 (100 nM) did not influence glucose-stimulated insulin secretion in mouse islets under control conditions (Suppl. Figure 3a). There was also no effect of PD-161570 on glucose-stimulated insulin secretion during glucolipotoxic culture (Suppl. Figure 3b). However, blocking FGFR attenuated the positive effect of FGF-23 against glucolipotoxicity (Fig. 3d). Next, we confirmed that the improved islet cell survival under glucolipotoxic conditions observed with FGF-23 was also depending on FGFR. PD-161570 (100 nM) and another FGFR inhibitor, PD-173074 (100 nM) [35], did not affect apoptotic cell death induced by glucolipotoxicity per se, but both FGFR blockers abolished the protective effect of FGF-23 (Fig. 3e).

### FGF-23 modulates electrical activity and cytosolic Ca^2+^ homeostasis in islets challenged by glucolipotoxicity

**Fig. 4:**
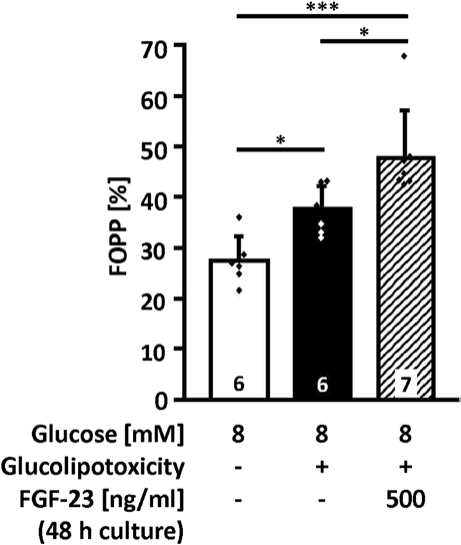
FGF-23 increases electrical activity under pathophysiological conditions. Electrical activity was determined by extracellular recordings of single islets placed on MEA chips. Electrical activity was induced by elevating glucose from 3 to 8 mM during the recording. After glucolipotoxic culture (25 mM glucose, 100 μM palmitate, 48 h), the fraction with electrical activity (fraction of plateau phase, FOPP) was elevated vs. control. Pre-treatment with FGF-23 potentiated this effect. Numbers in bars indicate the number of islets; *p < 0.05, ***p < 0.001

As glucose-mediated changes in electrical activity and [Ca^2+^]_c_ are key steps for the regulation of insulin release, we examined the effects of FGF-23 on these parameters. After 48-h pre-treatment in glucolipotoxic or standard medium, electrical activity was measured by extracellular recording on islets cultured on MEAs. For these measurements, glucose was elevated from 3 to 8 mM. The glucose concentration of 8 mM was chosen as it results in an electrical burst activity (slow waves) with a frequency that facilitates the detection of changes in both directions. The fraction of plateau phase amounted to about 30% after the acute change of glucose from 3 to 8 mM (Fig. 4, white bar). In islets challenged by glucolipotoxicity the fraction of plateau phase at 8 mM glucose was higher vs. control (Fig. 4, black vs. white bar). Co-culture with FGF-23 (500 ng/ml, 48 h) further elevated glucose-induced electrical activity (Fig. 4, hatched bar).

**Fig. 5:**
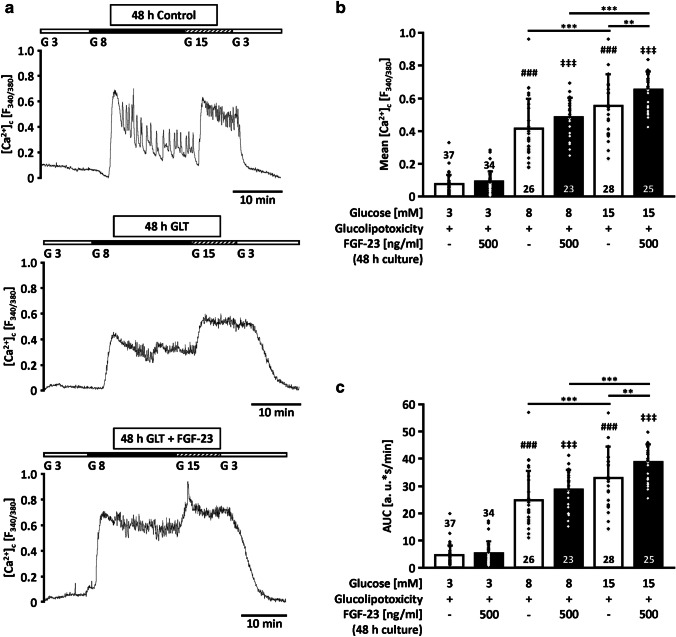
FGF-23 elevates [Ca^2+^]_c_ in response to glucose stimulation under glucolipotoxic conditions. The cytosolic Ca^2+^ concentration was measured after a culture period of 48 h in control or glucolipotoxic medium (25 mM glucose, 100 μM palmitate) supplemented with or without 500 ng/ml FGF-23. **a** The upper trace shows a representative experiment of an islet regularly oscillating when acutely stimulated with 8 or 15 mM glucose. Reducing glucose to 3 mM decreases [Ca^2+^]_c_ to a basal value. In the middle, the effect of glucolipotoxic culture (GLT) on [Ca^2+^]_c_ is illustrated. On the bottom, an experiment with islets after culture in glucolipotoxic medium supplemented with FGF-23 is shown. **b** Mean [Ca^2+^]_c_ and **c** AUC were increased by the application of 8 or 15 mM glucose. Co-culture with FGF-23 further enhanced mean [Ca^2+^]_c_ and the AUC when glucose was acutely elevated to 15 mM. Numbers in bars indicate the number of islets; **p < 0.01, ***p < 0.001, ^###^*p* < 0.001 vs. 3 mM glucose + glucolipotoxicity, ^‡‡‡^*p* < 0.001 vs. 3 mM glucose + glucolipotoxicity + 500 ng/ml FGF-23

With respect to Ca^2+^ signaling, reactivity of islets in response to different glucose concentrations was determined. In this series of experiments, islets were acutely stimulated by a rise of glucose concentration from 3 to 8, and 15 mM glucose (Fig. 5a) after a culturing period of 48 h in control or glucolipotoxic medium in the presence or absence of 500 ng/ml FGF-23. At the end of each experiment, glucose was lowered to 3 mM again to test for integrity of the coupling between glucose metabolism and Ca^2+^ influx. Mean [Ca^2+^]_c_ (Fig. 5b) and the area under the curve (AUC) (Fig. 5c) were analyzed. Glucolipotoxicity-challenged islets were still responsive to 8 and 15 mM glucose, respectively (white bars in Figs. 5b and c). The addition of FGF-23 to the glucolipotoxic culture medium had no significant impact on mean [Ca^2+^]_c_ and AUC when islets were acutely exposed to 3 or 8 mM glucose. By contrast, FGF-23 pre-treatment significantly elevated both parameters when the islets were stimulated by 15 mM glucose (G15: black vs. white bars in Fig. 5b and c).

### Calcineurin and JNK are decisive for the protective effects of FGF-23

**Fig. 6:**
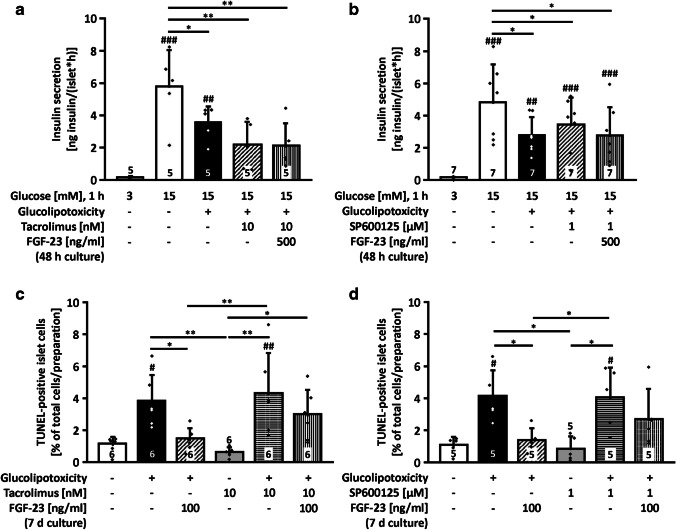
Calcineurin and JNK counteract the protection of FGF-23 against glucolipotoxic impairment. **a** Inhibition of calcineurin by tacrolimus (10 nM) during the 48-h culture period with glucolipotoxic medium and FGF-23 (500 ng/ml) abrogated the protective effect of FGF-23 on glucose-stimulated insulin release. **b** Inhibition of JNK by SP600125 (1 μM, 48 h) led to similar results. Of note, tacrolimus tends to augment the deleterious effect of glucolipotoxicity on glucose-stimulated insulin secretion. SP600125 did not alter the effect of glucolipotoxic culture per se. FGF-23 and inhibitors were not present during the steady-state incubation. **c** Inhibition of calcineurin by tacrolimus (10 nM) prevented the beneficial effect of FGF-23 (100 ng/ml) on glucolipotoxicity-mediated cell death (7 d). **d** Similar results were obtained for the inhibition of JNK by SP600125 (1 μM, 7 d). Glucolipotoxicity: 25 mM glucose, 100 μM palmitate (**a**, **b**); 33 mM glucose, 500 μM palmitate (**c**, **d**). Numbers in bars indicate the number of independent islet preparations; *p < 0.05, **p < 0.01, ^#^*p* < 0.05, ^##^*p* < 0.01, ^###^*p* < 0.001 vs. 3 mM glucose (**a**, **b**) or vs. control (**c**, **d**)

FGF-23 has been shown to activate the calcium- and calmodulin-dependent protein phosphatase calcineurin [37]. As calcineurin modulates gene expression and secretion of insulin [30], we investigated the effect of FGF-23 when calcineurin was blocked by tacrolimus (10 nM) in parallel during the 48-h culture in glucolipotoxic medium. FGF-23 lost its protective effect on glucose-stimulated insulin release under these conditions (Fig. 6a). FGF-23 is also known to interact with mitogen-activated protein kinases [2, 33]. The inhibitor SP600125 was used (1 μM) to test for potential involvement of c-Jun N-terminal kinase (JNK) [7]. Similar to the results obtained for tacrolimus, FGF-23 could not prevent the glucolipotoxicity-induced reduction of glucose-stimulated insulin release during inhibition of JNK signaling (Fig. 6b). As these results show that both pathways are involved in the mechanistic actions of FGF-23 on insulin secretion in murine islets, contribution of calcineurin and JNK to the anti-apoptotic effect of FGF-23 was elucidated. Addition of tacrolimus (10 nM) or SP600125 (1 μM) to control or glucolipotoxic medium for 7 d did not change the deleterious effect of permanently elevated glucose and lipid metabolism, but prevented the protective effect of 100 ng/ml FGF-23 (Fig. 6c and d, bars 3rd from left vs. bars 6th from left).

## Discussion

### FGF-23 as an endocrine regulator of metabolic disorders

FGF-23 was firstly described in 2000 as a polypeptide highly expressed in the ventrolateral thalamus [54]. On a lower level, it was also detected in the bone, liver, heart, lymph nodes, and (para)thyroid gland [21]. Osteocytes are thought to serve as major source for circulating FGF-23. Usually, plasma concentrations in healthy subjects are below 50 pg/ml but can be drastically elevated up to 1000-fold higher values, e.g., during progression of chronic kidney disease [11, 27]. At present, it is not clear whether an increase in FGF-23 plasma concentration is the cause or consequence of pathological processes. Elevated FGF-23 plasma concentrations were measured in patients with macroalbuminuric diabetic nephropathy [48], coronary artery disease [12], and other vascular diseases. Advanced glycation end products were reported to induce expression of FGF-23 [4]. Consequently, FGF-23 was suggested as a biomarker to identify persons at risk for diabetes-associated complications [10, 48]. In a recent examination, elevated serum levels of FGF-23 were reported in mice fed with a high-fat diet. The underlying pathway involved TNFalpha, linking regulation of FGF-23 generation to lipid homeostasis and inflammation [20]. Barely anything is known about the effect of FGF-23 on pancreatic tissue. For our in vitro study, we mainly used FGF-23 in a concentration of 100 ng/ml (single cell experiments) or 500 ng/ml (islets). The higher concentration (corresponding to ~ 20 nM FGF-23) was chosen to counterbalance reduced penetration of the protein through the capsule surrounding intact mouse islets.

Our data demonstrate for the first time that FGF-23 decreased glucose-mediated insulin release, whereas basal glucose-stimulated insulin secretion or islet cell apoptosis was not altered. Comparable effects were observed for FGF-21. Notably, FGF-15/19, which is linked to bile acid metabolism by acting via FGFR4 [26], also decreased glucose-stimulated insulin secretion in islets but, in addition, elevated apoptotic cell death. FGF-23 and FGF-15/19 both reduced the electrical activity of glucose-stimulated islets suggesting that an interaction with the classical stimulus-secretion cascade is responsible for the impaired secretory response. By contrast, FGF-21 did not influence this parameter. As FGF-21 was not in the focus of our current study, we did not further address the mechanism responsible for this discrepancy here.

### Protective function of FGF-23 in islets challenged by glucolipotoxicity

Chronically circulating elevated concentrations of glucose and lipids (gluco- and lipotoxicity) interfere with beta-cell function in numerous ways. A permanent supra-physiological substrate supply impairs mitochondrial function, stimulus-secretion coupling, and insulin release, accompanied by increased cell death [5, 13, 45]. In our in vitro model imitating an environment of glucolipotoxicity, the ability of islets to respond to glucose stimulation with an adequate change in [Ca^2+^]_c_ was examined. Changes in [Ca^2+^]_c_ are an important trigger for exocytosis [14]. After exposure to glucolipotoxicity, FGF-23 fosters the triggering effect of glucose on Ca^2+^ homeostasis and partly protects against the impairment of insulin release. The increase in mean [Ca^2+^]_c_ and AUC in response to glucolipotoxic culture coincided with an increase in electrical activity. This suggests that the classical stimulus-secretion coupling works up to the step of Ca^2+^ influx, but is not sufficient to compensate for the dramatic decrease in insulin content caused by glucolipotoxicity, which is only slightly affected by FGF-23. It is an important question whether potentiating insulin secretion is beneficial or drives beta-cell exhaustion in a long run. This depends on the status of the islets and on the extent of the effect. In principle, any strategy to preserve beta-cell function is desirable, if cells are not already too damaged. The latter one might be the case in patients suffering from severe insulin-deficient diabetes (SIDD), according to the novel classification system for T2DM subtypes [1]. To our knowledge, our study is the first to demonstrate that co-culture with FGF-23 reduced the pro-apoptotic effect of glucolipotoxicity, thereby rendering more cells glucose-responsive with respect to membrane depolarization and subsequent elevation of [Ca^2+^]_c_. Of note, FGF-15/19 also protects against glucolipotoxicity-mediated islet cell death, but is pro-apoptotic under physiological conditions. This switch between positive and negative influence cannot be explained yet, but is reminiscent of the farnesoid X receptor (FXR), that regulates the expression of FGF-15/19 and also changes its role in islet cells depending on the metabolic environment [42]. Anti-apoptotic effects of FGF-15/19 are also described for hepatocellular carcinoma cells under stress conditions [47]. There, the effect was linked to the Nrf2-cascade. Although this is far beyond the scope of our current study, involvement of Nrf2 is an interesting aspect as we previously described that pharmacological Nrf2 activation reduces insulin release under physiological conditions, whereas it is protective in metabolically challenged islets [43].

FGF-23 interacts with a large number of signaling pathways but with respect to extrarenal events most of them are only poorly characterized. By administration of two different FGFR inhibitors, the present study shows that the protective effects of FGF-23 depend on FGFR stimulation. The blockers antagonize the FGFR by inhibiting tyrosine kinase activity with different selectivity. PD-161570 inhibits FGFR1 [46], platelet-derived, and epidermal growth factor receptors, respectively [6]. PD-173074 shows high affinity for the FGFR family and, in addition, inhibits the vascular endothelial growth factor receptor. Crystal structure analysis confirmed a high degree of surface complementarity between PD-173074 and the hydrophobic ATP-binding pocket of FGFR1 underlining the potency of this compound [35].

Recently, a link between FGF-23 and the activation of calcineurin/NFAT via elevated Ca^2+^ influx was revealed for injury-primed renal fibroblasts [44]. Calcineurin was also shown to mediate FGF-23 signaling in the parathyroid gland [37]. Vice versa, inhibition of calcineurin by tacrolimus or cyclosporin A influenced FGF-23 expression in an osteoblast-derived cell line [3]. In insulin-secreting cells, the activity of calcineurin is increased by glucolipotoxicity [49]. It is suggested that the calcineurin/NFAT pathway supports adequate beta-cell function but that this may reverse and contribute to beta-cell failure under conditions of long-lasting metabolic “overload” [40]. To investigate whether calcineurin is involved in the compensatory protective effects of FGF-23 in our glucolipotoxic model, we used 10 nM tacrolimus for inhibition of phosphatase activation. We demonstrated in previous work, that tacrolimus, in contrast to cyclosporin A, per se does not reduce [Ca^2+^]_c_ in primary beta-cells even at a high concentration of 5 μM [15]. Our data clearly show that the protective effect of FGF-23 on insulin secretion and apoptotic cell death was lost in the presence of tacrolimus pointing to a crucial role of calcineurin in this scenario.

**Fig. 7:**
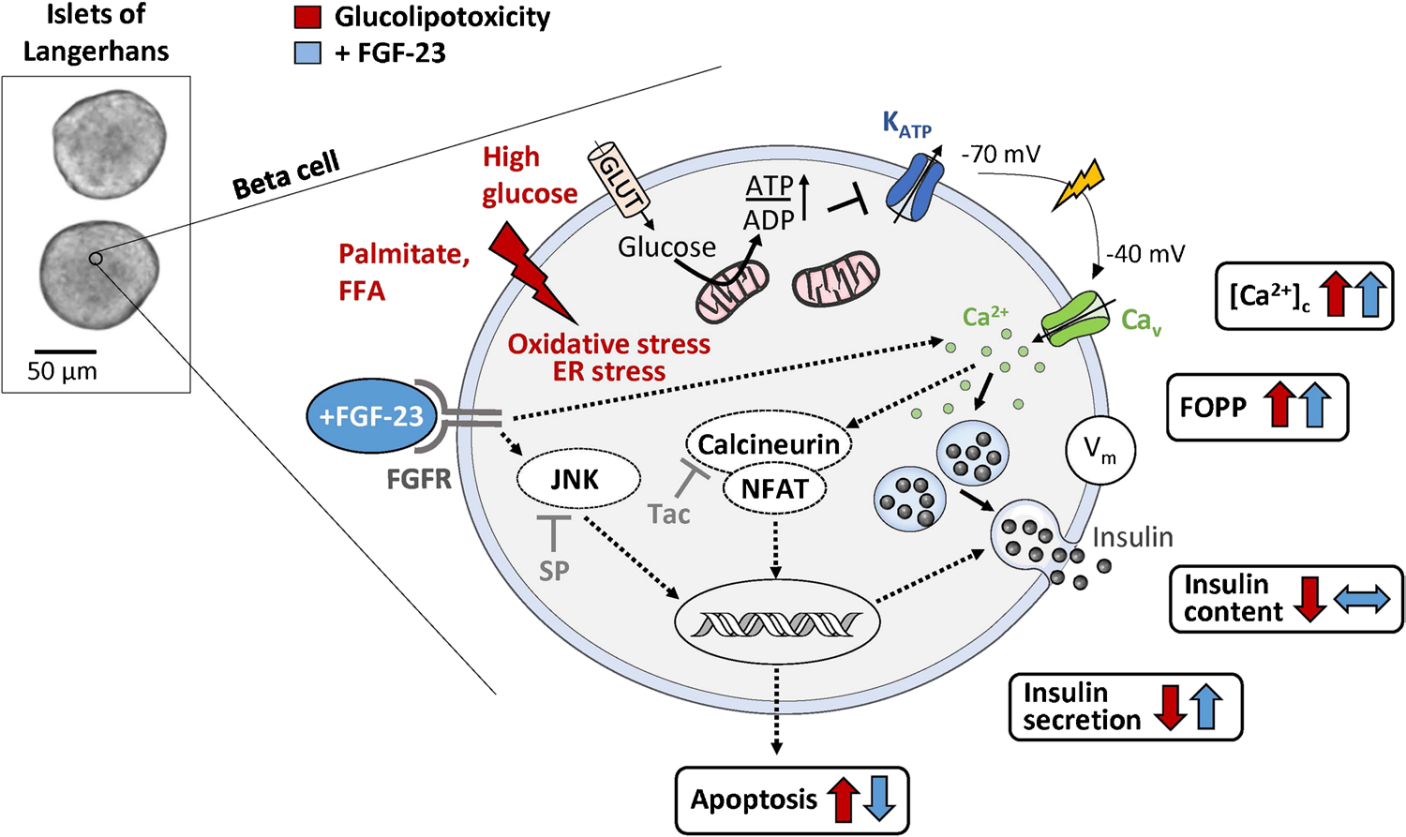
Summary of the effects of glucolipotoxicity and the protective influence of FGF-23. High glucose and palmitate concentrations induce beta-cell stress leading to a rise in apoptosis and a decrease in insulin content. This reduces glucose-stimulated insulin secretion. Compensatory mechanisms include an elevation of electrical activity and [Ca^2+^]_c_ influx. FGF-23 partly prevents beta-cell death and further augments membrane depolarization and Ca^2+^ influx. As a consequence, insulin secretion is enhanced in the presence of FGF-23 compared to glucolipotoxicity alone. Of note, the reduction in insulin content is slightly attenuated by FGF-23. Dotted lines indicate the proposed interaction partners of FGF-23 in beta-cells under glucolipotoxic conditions. [Ca^2+^]_c_, cytosolic Ca^2+^ concentration; FFA, free fatty acids; FGFR, fibroblast growth factor receptor; FOPP, fraction of plateau phase; JNK, c-Jun N-terminal kinase; SP, SP600125; Tac, tacrolimus.

Apart from calcineurin, other potential targets for FGF-23 in beta-cells are mitogen-activated protein kinases, e.g., extracellular signal-regulated kinase 1/2 (ERK1/2), protein kinase B/Akt, or c-Jun N-terminal kinases (JNK) [9, 33, 37, 40]. JNK is known to play a role in the regulation of various cellular stress responses. Among others, the kinase can interact with calcineurin-regulated gene transcription [31]. First, we tested whether inhibition of JNK alters the sensitivity of murine islet cells to glucolipotoxicity. The JNK blocker SP600125 did not change the influence of glucolipotoxicity per se indicating that the negative effects of high glucose combined with palmitate were not mediated by this kinase in our model. By contrast, inhibition of JNK abolished the protective effects of FGF-23 on insulin secretion and apoptosis. SP600125 was described to selectively target JNK [7] and by using a comparatively low concentration of 1 μM we exclude unspecific effects. At first glance, the result is astonishing, as blocking signaling cascades triggered by JNK is rather associated with positive effects on cell metabolism than with inhibition of protection [24, 25, 51]. However, activation of the JNK pathway can also be part of cell protective mechanisms or exert dual functions depending on the target tissue or metabolic status of the organism [38]. For example, experiments with liver-specific JNK1 knockout mice revealed that hepatic deletion of the kinase resulted in insulin resistance and hepatic steatosis [41]. Evidence linking positive effects of JNK to FGF-23 was provided for epithelial cells, where activation of JNK by FGF-23 was involved in the prevention of vitamin D_3_-mediated cell death [33]. The mechanism by which the FGF-23/JNK axis protects pancreatic beta-cells remains to be further investigated. Our results suggest that the potentiation of glucose-mediated Ca^2+^ influx and protection against cell death are key events for FGF-23-induced rescue mechanisms. The proposed interactions for FGF-23 in beta-cells impaired by glucolipotoxicity are summarized in Fig. 7.

Unlike FGF-23, FGF-21 has already been characterized in more detail with respect to its influence on the endocrine pancreas and also on glucose homeostasis in vivo [39, 52]. Data of our study is based on in vitro experiments, and up to now, we can only hypothesize about the impact of FGF-23 on glycemic control of the whole organism. Compared to FGF-21, our results show that FGF-23 seems to be even more effective in reducing apoptotic islet cell death in a glucolipotoxic environment. Wente et al. [52] reported improvement of insulin release by ~ 1100 ng/ml (50 nM) FGF-21 in rat islets treated with palmitate and in islets of db/db mice. They did not investigate involvement of JNK but identified an interaction with another MAPK signaling cascade, ERK1/2-Akt, as crucial for the action of FGF-21 on insulin content. A recently published paper by Pan et al. suggests PI3K/Akt signaling as a critical mediator for beneficial effects of FGF-21 on insulin secretion. They postulated that FGF-21 promotes insulin gene transcription factors and soluble N-ethylmaleimide-sensitive factor attachment protein receptor (SNARE) proteins by a yet unidentified mechanism [39]. Whether and to which extent FGF-23 and FGF-21 share common protective pathways needs to be identified.

In summary, we provide first evidence for a regulatory role of FGF-23 in pancreatic islets. FGF-23 diminishes the pathological changes in murine islets cells induced by high glucose and high lipid supply. These effects are mediated (a) by enhancing the response of [Ca^2+^]_c_ which fosters insulin release upon stimulation with glucose and (b) by protecting islet cells against cell death. These protective mechanisms depend on pathways involving calcineurin and JNK.

## Supplementary information

Below is the link to the electronic supplementary material.

Supplementary file1 (DOCX 151 KB)

## Data Availability

Not applicable.
