# Supplementary material for: FGF-23 protects cell function and viability in murine pancreatic islets challenged by glucolipotoxicity
Source: Pflugers Arch. 2022 Nov 28;475(3):309–22. doi: 10.1007/s00424-022-02772-x (PMC9908675; doi:10.1007/s00424-022-02772-x)
Supplement: Supplementary file 1 — Supplementary file1 (DOCX 151 KB) [file 424_2022_2772_MOESM1_ESM.docx]

**Supplementary material**

**Pflügers Archiv – European Journal of Physiology**

**FGF-23 protects cell function and viability in murine pancreatic islets challenged by glucolipotoxicity**

Betina Pajaziti^1^, Kenneth Yosy^1^, Olga V. Steinberg^1^, and Martina Düfer^1^*

^1^University of Münster, Institute of Pharmaceutical and Medicinal Chemistry, Dept. of Pharmacology, Corrensstraße 48, 48149 Münster, Germany

Short title: Influence of FGF-23 on beta-cell function and viability

*Corresponding author: Prof. Dr. Martina Düfer

University of Münster, Institute of Pharmaceutical and Medicinal Chemistry, Dept. of Pharmacology, Corrensstraße 48, 48149 Münster, Germany

Phone: (+49) 251 83 33339; Fax: (+49) 251 83 32144

martina.duefer@uni-muenster.de

ORCID ID: 0000-0003-1572-9235

**Supplementary Figure 1**


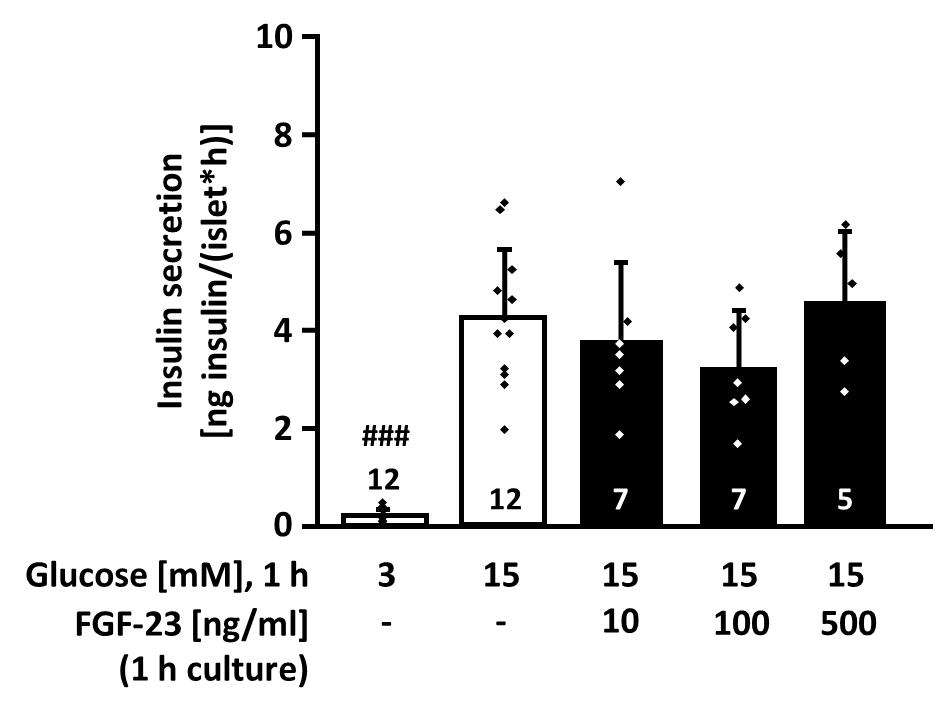


**Suppl. Fig. 1** Acute administration of FGF‑23 for 1 h does not affect glucose-stimulated insulin release

Murine islets were incubated for 1 h with or without FGF-23 before determination of insulin secretion by a steady-state incubation (acute, 1 h). Glucose-stimulated insulin release (15 mM glucose) was not affected by the preincubation with 10, 100 or 500 ng/ml FGF-23. In these experiments FGF-23 was not present during the acute determination of glucose-stimulated insulin secretion. Numbers in bars indicate the number of independent islet preparations; ^###^p<0.001 vs. all other conditions.

**Supplementary Figure 2**


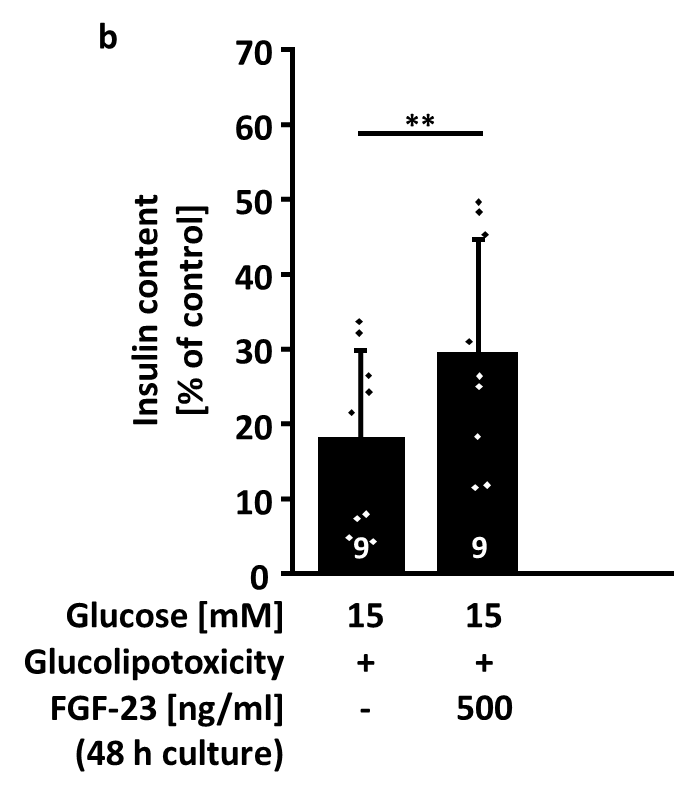
**
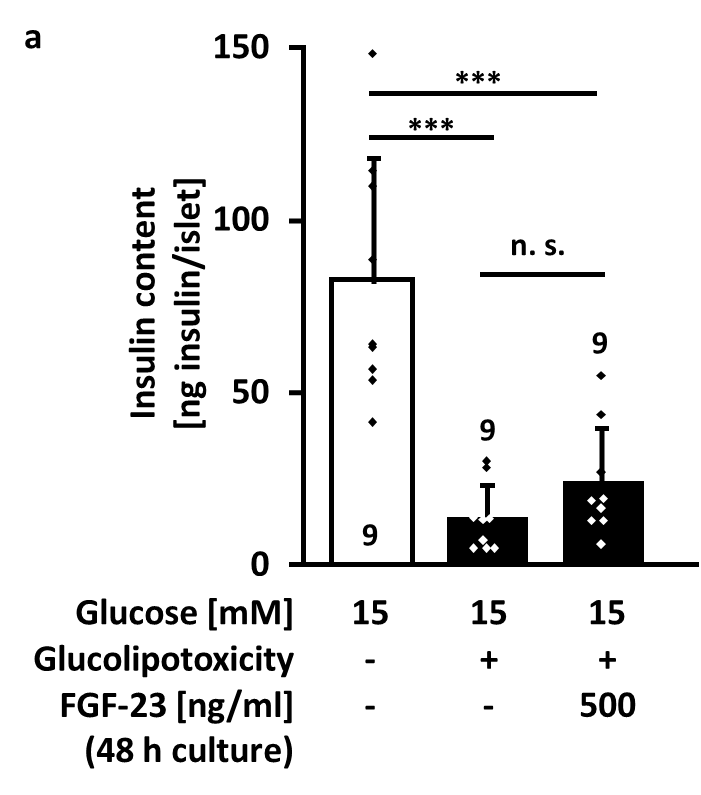
**

**Suppl. Fig. 2** FGF-23 only slightly affects changes in insulin content in murine islets during glucolipotoxic culture

Chronic treatment of murine islets with glucolipotoxic medium for 48 h lowered insulin content. Co-culture with 500 ng/ml FGF‑23 slightly reduced this effect. In (a) absolute values are illustrated, in (b) data are normalized to the insulin content determined under control conditions in each experiment. Glucolipotoxicity: 25 mM glucose, 100 µM palmitate. Numbers in bars indicate the number of independent islet preparations; ***p<0.001, **p<0.01, n. s.: not significant.

**Supplementary Figure 3**

**
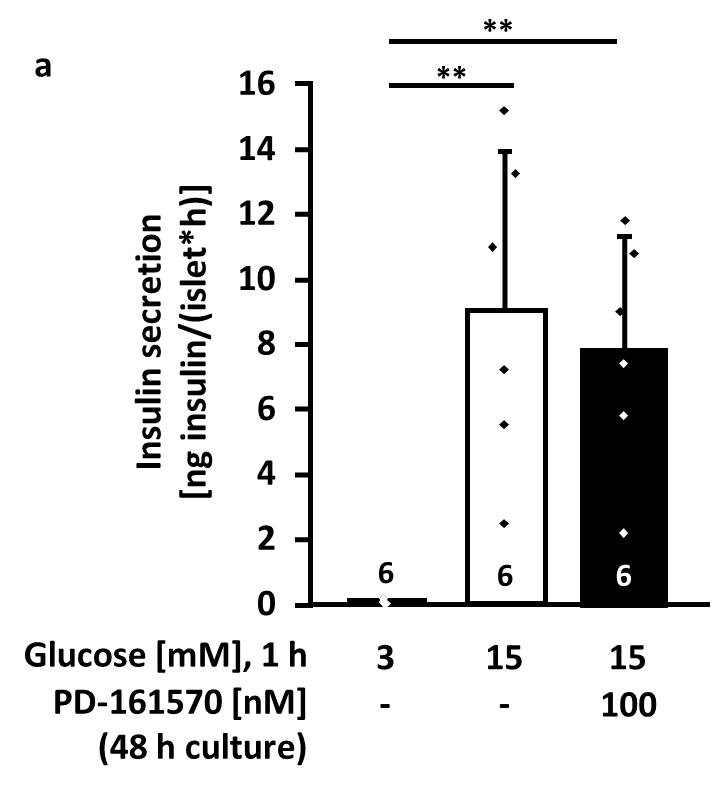
**

**
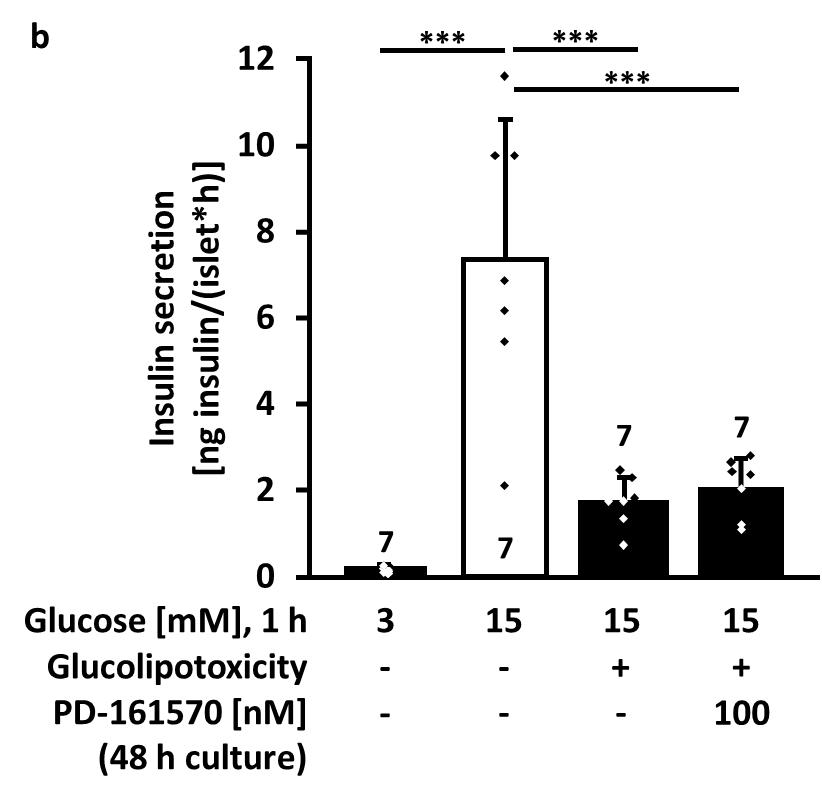
**

**Suppl. Fig. 3** PD-161570 does not influence glucose-stimulated insulin secretion in murine islets in control or glucolipotoxic medium after culture for 48 h

Islets were cultured for 48 h in (a) control medium with or without 100 nM PD-161570 or (b) in glucolipotoxic medium with or without 100 nM PD-161570. PD-161570 did not influence glucose-stimulated insulin secretion (induced by 15 mM glucose for 1 h) in mouse islets after culture under standard or glucolipotoxic conditions. Glucolipotoxicity: 25 mM glucose, 100 µM palmitate. Numbers in bars indicate the number of independent islet preparations; **p<0.01, ***p<0.001.
